# Multimodal Utility Data for Appliance Recognition: A Case Study with Rule-Based Algorithms

**DOI:** 10.3390/s26020527

**Published:** 2026-01-13

**Authors:** Arkadiusz Orłowski, Krzysztof Gajowniczek, Marcin Bator, Robert Budzyński

**Affiliations:** 1 Instytut Informatyki Technicznej, Szkoła Główna Gospodarstwa Wiejskiego, 02-776 Warszawa, Poland; krzysztof_gajowniczek@sggw.edu.pl (K.G.); marcin_bator@sggw.edu.pl (M.B.); robert_budzynski@sggw.edu.pl (R.B.); 2 Wydział Cybernetyki, Wojskowa Akademia Techniczna, 00-908 Warszawa, Poland

**Keywords:** non-intrusive load monitoring, multimodal sensing, smart metering, appliance recognition, rule-based algorithms

## Abstract

Appliance recognition from aggregate household measurements is challenging under real deployment conditions, where multiple devices operate concurrently and sensor data are affected by imperfections such as noise, missing samples, and nonlinear meter response. In contrast to many studies that rely on curated or idealized datasets, this work investigates appliance recognition using real multimodal utility data (electricity, water, gas) collected at the building entry point, in the presence of substantial uninstrumented background activity. We present a case study evaluating transparent, rule-based detectors designed to exploit characteristic temporal dependencies between modalities while remaining interpretable and robust to sensing imperfections. Four household appliances—washing machine, dishwasher, tumble dryer, and kettle—are analyzed over six weeks of data. The proposed approach achieves reliable detection for structured, water-related appliances (22/30 washing cycles, 19/21 dishwashing cycles, and 23/27 drying cycles), while highlighting the limitations encountered for short, high-power events such as kettle usage. The results illustrate both the potential and the limitations of conservative rule-based detection under realistic conditions and provide a well-documented baseline for future hybrid systems combining interpretable rules with data-driven adaptation.

## 1. Introduction

The rapid deployment of smart metering technologies has reshaped the monitoring of electricity, water, and gas consumption. Initially conceived for billing and basic demand tracking, metering infrastructures now support energy efficiency, sustainability, and intelligent control. The concept of non-intrusive load monitoring (NILM) [1] demonstrated that device-level usage can be inferred from aggregate power data, while multimodal sensing extends this idea by combining electricity with water and gas to disambiguate overlapping signatures and improve robustness.

Contemporary challenges such as climate change, rising energy costs, and policy directives on sustainability have reinforced the need for fine-grained feedback. Accurate recognition of appliances enables demand-side management, fault detection, and behavior-aware feedback. Multimodal metering offers richer evidence than electricity-only systems and provides a bridge between technical sensing and human-centered sustainability goals. At the same time, policy frameworks such as the European Union’s 2023 Energy Efficiency Directive  [2] promote advanced metering and consumer empowerment. To be actionable, sensing must be trustworthy, privacy-aware, and explainable. Transparent algorithms are increasingly preferred to black-box models, especially in regulated environments.

As NILM research has matured, many approaches have explicitly addressed non-ideal sensing conditions through modeling choices, preprocessing strategies, or hybrid system designs. In the present study, we adopt a complementary perspective by treating deployment-related imperfections—such as sensor noise, missing samples, timestamp discontinuities, and device interference—as an integral part of the problem and by documenting their impact explicitly rather than abstracting them away.

Our contributions are threefold: (1) documentation of sensing challenges in multimodal data, (2) definition of behavioral rule-based detectors for four household appliances, and (3) quantitative and qualitative validation, including guidance for future hybrid systems.

In addition to these technical goals, this work can be viewed as an attempt to bridge two cultures of thinking about energy data. On the one hand, large-scale policy documents motivate metering mainly through aggregate indicators and national targets; on the other, everyday household reality is granular, noisy, and strongly context-dependent. By explicitly connecting a very small, concrete case study with the broader landscape of EU-level efficiency policies [2], we aim to show how even modest, well-documented experiments can inform the design of monitoring tools that have a realistic chance of adoption beyond the laboratory.

This study is conceived as a practical case study rather than a benchmark-oriented methodological contribution. Its primary objective is to investigate conservative, interpretable appliance identification under fully aggregated, real-world sensing conditions, where robustness and operational usefulness are prioritized over optimized performance metrics.

Finally, the choice of a rule-based core is deliberate. Instead of using a complex model and subsequently explaining it, we begin with interpretable rules and ask how far such a minimalist approach can be pushed before more sophisticated tools become necessary. This question is not only methodological but also practical: it touches on the long-term maintainability of NILM systems, the ability of non-expert stakeholders to audit decisions, and the possibility of combining simple detectors with more advanced analytics in future work.

## 2. Background and Related Work

Non-intrusive load monitoring (NILM) research began with Hart’s seminal work [1], which demonstrated that appliance-level activity can be inferred from aggregate electrical measurements by identifying characteristic events such as edges and steady-state levels. Subsequent surveys [3] systematized early approaches, ranging from edge detection and harmonic analysis to probabilistic models and neural networks. The availability of public datasets and toolkits, including REDD [4] and NILMTK [5], enabled systematic comparison of algorithms and accelerated methodological development.

In recent years, deep learning approaches have come to dominate the NILM literature [6]. These methods are capable of learning complex appliance signatures and temporal dependencies, often achieving high performance on benchmark datasets. However, their effectiveness typically relies on large, carefully curated training data and extensive preprocessing. In contrast, real residential sensing environments are characterized by noise, missing samples, sensor saturation, timestamp discontinuities, and uncertain or incomplete labels, all of which complicate direct deployment of purely data-driven models.

Multimodal sensing has been proposed as a way to mitigate some of these challenges. By combining electricity with complementary modalities such as water and gas, it becomes possible to exploit cross-modal correlations and disambiguate overlapping electrical signatures. The AMPds2 dataset [7] is a notable example, integrating electricity, water, and gas measurements to reveal household activity patterns. Other reviews and surveys [8,9] emphasize the role of multimodal fusion in improving robustness and contextual awareness in smart environments. Related work in water metering [10] further demonstrates how fine-grained consumption analysis can support sustainability and demand management goals. Earlier applied studies by Ząbkowski, Bator, and Orłowski [11] explored practical aspects of smart metering architectures, providing empirical foundations relevant to the present work.

Beyond algorithmic design, recent literature increasingly stresses the importance of evaluation methodology. As discussed by Pereira et al. [12], reported NILM performance is strongly influenced by dataset characteristics, class imbalance, and the choice of metrics. Many studies rely on aggregate scores computed over relatively short or curated recordings, which may obscure failure modes visible in long-term, real-world deployments. Such deployments expose additional issues, including concept drift, heterogeneous user behavior, and incomplete reference labeling, which are not well captured by single summary statistics. This has motivated the use of case-based reporting and visual inspection of time series alongside conventional scalar metrics.

Several recent surveys synthesize these concerns in broader methodological overviews. Peng et al. [6] review deep learning approaches for NILM, highlighting both their representational power and their dependence on data quality. Cruz-Rangel et al. [13] focus on online NILM, identifying challenges related to real-time operation, delayed labels, and computational constraints. Saleem et al. [14] and Huzzat et al. [15] provide comprehensive reviews of traditional, machine learning, and deep learning techniques, noting current trends toward hybrid and multimodal architectures while emphasizing persistent difficulties in generalization across households and operating conditions.

Another important line of research concerns explainability and user acceptance. As NILM outputs are increasingly proposed as inputs for user feedback, building management, or policy-related decisions, the ability to trace detections back to transparent and auditable criteria becomes critical. Rule-based systems and hybrid architectures, where explicit rules serve as an interpretable layer alongside data-driven components [8,9], are therefore attracting renewed interest.

In contrast to the above works, which primarily aim at benchmarking and improving predictive performance of learning-based models, the present study adopts a deliberately complementary perspective. Rather than proposing a new learning algorithm, we investigate a conservative, rule-based identification scheme applied to fully aggregated, real-world multimodal data affected by sensing imperfections and uninstrumented background activity. Our contribution lies in documenting how interpretable rules behave under realistic deployment conditions, clarifying their strengths and limitations, and providing a well-defined baseline for future hybrid NILM systems that may combine transparent rule logic with data-driven adaptation.

## 3. Experimental Setup and Data

The dataset originates from a residential testbed equipped with electricity, water, and gas sensors. Electricity power readings were sampled at 50 Hz (stored every 20 ms), while water and gas counters updated every second with 1 L resolution. The observation period covered 39 days of continuous data. Reference on/off annotations were available for seven appliances, with reliable references for four: washing machine, dishwasher, tumble dryer, and kettle.

Several imperfections were encountered: non-monotonic timestamps caused by counter resets, spurious zero readings, nonlinear meter response at high loads, and missing or swapped reference labels. Data preprocessing involved timeline alignment, removal of impossible zeros, and empirical correction of nonlinearity. These imperfections are representative of real deployments and motivate the rule-based robustness analysis.

From a practical perspective, the household under study can be viewed as a small but representative example of urban European dwellings. The appliances considered are common in many countries, and their operating profiles reflect everyday use rather than scripted laboratory scenarios. This makes the dataset particularly useful for testing whether a simple rule-based framework can handle irregular usage (e.g., cycles interrupted by the user, atypical loading of the washing machine) without being overfitted to an artificially clean regime.

It is also worth emphasizing that the multimodal infrastructure was not originally designed specifically for NILM research but emerged from a broader smart metering pilot [11]. As a result, the data reflect the sort of “warts and all” imperfections one should expect in deployment: occasional outages, reboots, logging glitches, and irregular annotation density. The present study uses these characteristics as a stress test for detector design, rather than treating them as nuisances to be removed.

The raw data supplied for analysis differed from the format originally assumed. Initially, the plan was to work with separate daily files for each utility (electricity, water, gas), each containing a full 24 h record. In practice, the provider (Vedia SA) delivered compressed files, each covering approximately one hour of data and containing interleaved records from all meters. Each line in such a file corresponds to a single reading and consists of comma-separated fields: a meter identifier beginning with the dollar symbol (e.g., $1), an integer timestamp in units of 0.01 s from system start-up, and one or more integer fields whose interpretation depends on the meter type. For electricity, the third field stores instantaneous active power in watts; for water and gas, the counters are updated once per second with 1 L resolution by means of camera-based devices attached to the mechanical dials of the meters (image analysis of the rotating digit).

An additional channel contained binary reference information indicating the activity or inactivity of seven selected devices in the household. In parallel, there was an experimental attempt to capture acoustic context using a dedicated audio recorder, but these sound data were not used in the present analysis.

The first class of problems became visible when inspecting the timestamp field: in some fragments the time value suddenly decreased instead of monotonically increasing, indicating counter resets or mis-ordered records. A simplified illustration is given by the following extract from a real file:

$1 38575118 356

$1 38575120 356

$1 38575122 356

$oC2 38575117 2591 0

$1 38575123 0

$1 34717612 0

$1 30860102 0

$1 27002591 0

$1 23145081 0

$1 19287571 0

$1 15430060 358

$1 11572550 357

$1 7715039 357

$1 3857529 357

$r47 38575134 0 0

$o9C 38575139 25 0

$1 38575145 357

$1 38575147 357

Here, after a reading with timestamp 38575123, the subsequent entries for the same meter ID show time values that jump to much smaller numbers and then gradually increase again. The number of missing samples between 38575123 and 38575145 is relatively small, but the raw timestamps clearly violate the expected monotonicity. In addition, electricity readings sometimes take the value zero in situations where the aggregate household load could not realistically be zero; these zeros frequently coincide with timestamp anomalies but also occur elsewhere. In many cases, the first non-zero value after such a sequence is smaller than subsequent readings, suggesting that it is also corrupted. All these effects contribute to effectively missing or distorted data segments.

A second, more subtle problem was detected only after detailed analysis: the electrical power readings exhibit apparent nonlinearity at high loads. When inspecting sequences corresponding to washing-machine drum rotations, it became evident that the incremental power difference between “background” and “active” states depended strongly on the absolute load level. In the raw traces, three bands of operation can be distinguished, roughly in the ranges 300–1500 W, 2500–3000 W, and 4300–4500 W. In each band, the drum performs the same mechanical work and should therefore induce a similar increase in power. Empirically, however, the observed increments were around 1200 W, 500 W, and 200 W, respectively. This behaviour made it impossible for the original washing-machine detector to rely on a single characteristic step height.

**Figure 1:**
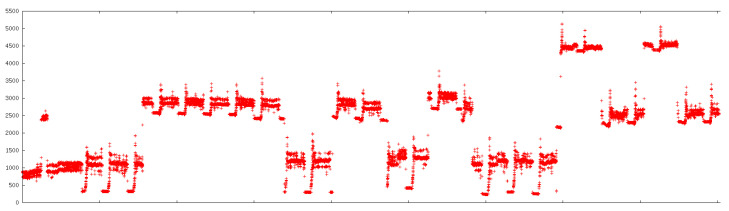
Example of apparent nonlinearity in the raw active power measurements for washing-machine drum rotations. The effective step height depends strongly on the absolute load band.

**Figure 2:**
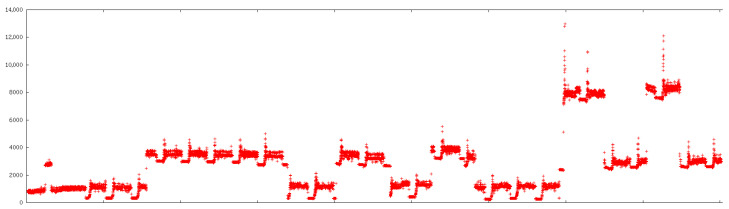
The same time interval as in Figure 1 after applying an empirical nonlinearity correction function f(x). The effective step heights become more comparable across load bands.

Figure 1 illustrates such a raw power trace with repeated drum-rotation patterns, while Figure 2 shows the same interval after applying an empirical nonlinearity correction. The goal of the correction was not to reconstruct the exact missing physics, but to make the effective power increments across bands at least comparable so that a single rule could capture the behaviour.

The correction was implemented via a piecewise function f(x) that maps the measured power *x* to a compensated value:

(1) $$f\left(x\right)=\left\{\begin{array}{cc} x, & x<1800,\\ -\frac{\log\;\left(1-\frac{x}{5500}\right)}{0.00022}, & 1800\leq x<5500,\\ -\frac{\log\;\left(1-\frac{5499}{5500}\right)}{0.00022}, & x\geq 5500. \end{array}\right.$$

For low loads the readings are left unchanged; for higher loads a logarithmic mapping is used to counteract saturation. The correction does not perfectly equalize all increments but brings them into a regime where the drum-rotation detector can operate reliably. Later clarifications from Vedia engineers indicated that the nonlinearity was likely caused by the way the clamp-on current sensor was attached near the fuse box and by saturation effects in the associated transformer.

Another important limitation concerned the reliability of reference labels. For example, the microwave oven used in the household allows the user to set the operating time in multiples of 30 s, yet the reference data contained only very short entries of one to five seconds. For the vacuum cleaner and iron, reference logging was implemented via a shared extension cord with two sockets, but in practice the devices were occasionally plugged into the wrong socket. As a result, the labels sometimes indicated the iron when in fact the vacuum cleaner was running, or vice versa. Automatic use of these labels would therefore require manual cross-checking of the corresponding power traces to establish which device was actually active.

Finally, some appliances of clear interest for NILM were present in the household but not instrumented with reference sensors. In particular, the electric hob, oven, and refrigerator draw amounts of power comparable to the four studied devices and can therefore interfere with their detection. Due to limited access to the sockets feeding these appliances, no reference signals were recorded for them. From the point of view of the present study, this is both a limitation and an opportunity: explicitly modelling and subtracting such “background” large loads would almost certainly improve the clarity of the residual signal for the remaining devices.

## 4. Methods

We implemented parameterized rule-based detectors encoding characteristic behavioral patterns for each device. Parameters were tuned specifically to the household dataset, balancing detection sensitivity and false alarms.

Let us now clarify the internal logic and rules underlying the proposed detectors. The description below is intentionally procedural and conceptual rather than code-level, in order to emphasize interpretability and to make explicit how detection decisions are formed from fully aggregated measurements.

The proposed rule-based scheme operates as a hypothesis–verification process applied to total household electricity, water, and gas consumption. At the input level, only aggregate signals measured at the building entry point are available. Importantly, numerous other appliances and activities—such as showering, tap usage, cooking, refrigeration, or electric hob operation—occur concurrently and are not instrumented with reference sensors. Consequently, candidate appliance signatures must be identified in the presence of substantial and structured background activity.

The detection process begins with the generation of candidate hypotheses based on characteristic temporal dependencies between modalities, in particular the co-occurrence of changes in water usage and electrical power (rising and falling edges). Such temporal patterns indicate the potential activation of a given appliance but are not yet treated as definitive detections. At this stage, hypotheses are deliberately permissive in order to avoid prematurely rejecting true events in noisy or imperfect data.

Each candidate hypothesis is subsequently subjected to device-specific verification. This step tests whether the observed signals satisfy empirical constraints derived from typical appliance behavior, including minimum and maximum power levels, temporal persistence, and internal structure of the activation (e.g., repeated drum rotations, heating phases, or characteristic idle intervals). Consistency across modalities is also required; for example, water intake without a corresponding electrical signature, or vice versa, leads to hypothesis rejection.

If one or more constraints are violated, the hypothesis is falsified. This explicit falsification mechanism reflects a conservative design philosophy: in ambiguous situations, it is preferable to miss borderline activations than to generate spurious detections that would undermine interpretability in a real deployment context. Equation (2) provides a simplified abstraction of this logic by expressing activation in terms of threshold and duration; in practice, the implemented rules consist of multiple such conditions arranged in temporal sequences that reflect appliance-specific operational phases.

When a hypothesis is verified, the estimated contribution of the corresponding appliance is subtracted from the aggregate electrical signal before subsequent detectors are applied. This sequential processing reduces interference between appliances and limits double counting when multiple devices operate simultaneously. The order of detector application follows appliance complexity: longer and more structured devices (washing machine, dishwasher, tumble dryer) are processed first, while short, high-power events (kettle) are evaluated on the residual signal.

It should be emphasized that the adopted rules are not intended to exhaustively model all possible appliance behaviors. Rather, they encode a conservative subset of characteristic patterns that remain recognizable under imperfect sensing conditions, including timestamp discontinuities, spurious zero readings, and nonlinear meter response. The resulting detections should therefore be interpreted as robust and explainable indicators rather than as optimal or exhaustive classifications.

Given the relatively simple and interpretable structure of the proposed rules, the detection pipeline is described procedurally rather than through a separate flowchart.

Based on the general detection logic outlined above, the following paragraphs describe the appliance-specific rules and parameters used for each of the analyzed devices.

Kettle. Short high-power bursts were identified by threshold edges near 1600 W (on) and 1400 W (off), with boiling times between 3 s and 12 min. Edge checks were validated over short sliding windows to account for sensor slew. In practice, the rise and fall of power are not instantaneous at the sampling resolution of 20 ms; therefore, the algorithm compares samples separated by two time steps (1/25 s) for kettle switch-on and five time steps (1/10 s) for switch-off, rather than relying solely on adjacent samples.

Washing machine. Detection combined electricity and water signals: initial water intake ≥12 L within 120 s, followed by rinses of ∼20 L and characteristic drum rotation cycles (≈16.4 s active, 4 s idle) with power steps ≥800 W. The algorithm tolerated minor deviations, allowing up to four consecutive out-of-range samples. Individual rotation cycles could occasionally be missed without invalidating the overall detection. A spin phase of roughly 450 s was also expected near the end of the cycle.

Dishwasher. Recognized through repeated water intakes (≥10 L over 60 s) and heating phases (∼1500 W) separated by temporal gaps (30–65 min between heating phases). Heating typically occurred within the first 10 min and again near 75–85 min of operation. The total cycle length was constrained to lie within a window derived from the time between the first and second water-intake phases plus an additional margin.

Tumble dryer. Identified by periodic rotation signatures of ≈251 s with initial power step around 1400 W. Partial cycle detections were also logged: if the characteristic rotation pattern was present for part of the run, this was counted as a successful detection, as the dataset did not contain isolated reference traces for this appliance.

The detection logic is based on thresholding of measured current (or power), combined with persistence verification to avoid false triggers caused by short transients. Device-specific parameters were established empirically during preliminary trials. Each appliance can be viewed as being associated with a logical rule of the form

(2) $$A_{i}\left(t\right)=\left\{\begin{array}{cc} 1, & \mathrm{if}\;I\left(t\right)>T_{i}\;\mathrm{for}\;\mathrm{at}\;\mathrm{least}\;\tau_{i}\;\mathrm{seconds},\\ 0, & \mathrm{otherwise}, \end{array}\right.$$

where $T_{i}$ is the current (or power) threshold and $\tau_{i}$ the minimum activation duration for device *i*. Equation (2) should be understood as a high-level abstraction of the rule-based approach rather than a complete enumeration of all implemented rule checks and temporal sequences. In practice, more elaborate temporal patterns (sequences of such conditions with specific intervals) are used, but the basic logic remains the same.

Additional corrections were applied to handle simultaneous operation and overlapping signals. A validation step excluded implausible activations (e.g., dishwasher and kettle within 10 s) to prevent double counting. The overall processing order followed the complexity of the appliances: detectors for more structured, longer-lasting devices (washing machine, tumble dryer, dishwasher) were applied first, and their estimated contributions were subtracted from the aggregate power signal. The kettle detector, designed for short high-power bursts, operated on the residual. This ordering reflects the intuition that large, structured loads should be accounted for before attempting to interpret shorter transients.

In designing these rules, we followed a conservative philosophy: it was preferable to miss borderline events than to generate a large number of spurious detections. In ROC terminology [16,17], this corresponds to operating in a region of relatively high specificity, accepting some loss of sensitivity in exchange for clearer, more defensible outputs. This choice reflects the kind of trade-off that would be made in a deployed system where false alarms carry real costs for users or operators.

Another methodological consideration concerns the degree of coupling between device models. Our current implementation treats the rules as largely independent, with only simple conflict-avoidance heuristics. An alternative design would be to introduce stronger joint constraints (e.g., disallowing certain combinations of devices or enforcing power balance at the aggregate level). We deliberately postpone such extensions to future work, as they complicate interpretation and tuning; nonetheless, the present modular design is compatible with such enhancements.

## 5. Evaluation and Results

The detectors were evaluated using standard binary classification metrics: accuracy, sensitivity (recall), and specificity. These indicators quantify the proportion of correct detections, the ability to identify true events, and the ability to avoid false alarms, respectively. Formally, for a given device we define

(3) $$\begin{array}{cc} \mathrm{Accuracy} & =\frac{n(\mathrm{TP})+n(\mathrm{TN})}{n(\mathrm{TP})+n(\mathrm{FP})+n(\mathrm{FN})+n(\mathrm{TN})}, \end{array}$$

(4) $$\begin{array}{cc} \mathrm{Sensitivity} & =\frac{n(\mathrm{TP})}{n(\mathrm{TP})+n(\mathrm{FN})}, \end{array}$$

(5) $$\begin{array}{cc} \mathrm{Specificity} & =\frac{n(\mathrm{TN})}{n(\mathrm{FP})+n(\mathrm{TN})}, \end{array}$$

where n(TP), n(FP), n(FN), and n(TN) denote the number of true positives, false positives, false negatives, and true negatives, respectively, under a given notion of “event”.

In an earlier phase of the project, these metrics were applied to classifiers based on statistical features computed over fixed windows of the aggregate signal. Table 1 summarizes an example from that stage (reported in an internal document on feature relevance). Although the numerical values of accuracy and sensitivity appear high for some devices, visual inspection of the underlying time series revealed that the corresponding detections were largely unusable in practice, especially for devices with short activation periods such as the kettle.

As the last row indicates, it is possible to obtain very high apparent accuracy for the kettle while failing to detect any real activations (sensitivity equal to zero). This illustrates a well-known but often overlooked issue: in highly imbalanced problems, metrics dominated by the large number of negative instances can seriously misrepresent practical usefulness. Consequently, in the present study we treat scalar metrics as a partial summary only and complement them with systematic visual evaluation of daily plots.

All outcomes were visually inspected by an expert using daily signal plots. Each plot shows results for one specific day from the data collection period. In all figures, the legend refers to the original Polish file names used in the implementation: pralka.txt (washing machine), zmywarka.txt (dishwasher), suszarka.txt (tumble dryer), and czajnik.txt (kettle). If a particular file name is absent in a figure, this simply means that the corresponding device was not used on that day. In each plot, the horizontal axis (OX) represents time in minutes from the beginning of the day, while the vertical axis (OY) shows the classification outcome for the following devices (from top to bottom): dishwasher, tumble dryer, washing machine, and kettle. At the very bottom of each plot, we additionally indicate abnormal behaviour of the current sensors (bledy_elec.txt). For each device, up to four indicators can appear: missed detections (FN, blue), false alarms (FP, red), correct detections (TP, green), and reference labels from the manually curated ground-truth annotations (grey).

In this section, we illustrate five representative days (Figure 3, Figure 4, Figure 5, Figure 6 and Figure 7); additional selected days covering the remaining observation period are provided in Appendix A (Figure A1, Figure A2, Figure A3, Figure A4, Figure A5, Figure A6, Figure A7, Figure A8, Figure A9, Figure A10, Figure A11, Figure A12 and Figure A13), in order to document the consistency of the detectors across different operating conditions.

Across six weeks of data:

- Washing machine: 22/30 cycles detected (no false positives);
- Dishwasher: 19/21 detected (5 false positives);
- Tumble dryer: 23/27 detected (partial cycles counted);
- Kettle: 61 true positives, 72 false positives, 150 false negatives.

**Figure 3:**
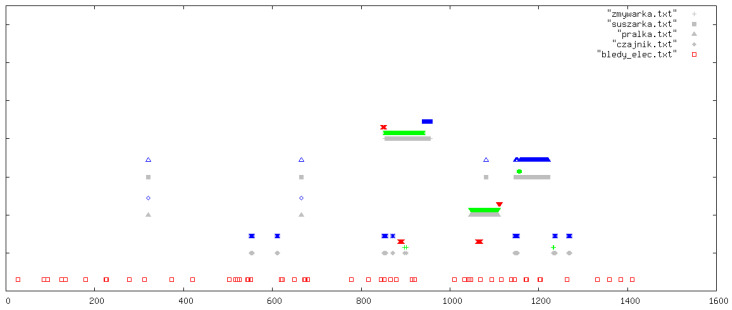
Example detections for one selected day. The horizontal axis (OX) represents time in minutes from the beginning of the day. The vertical axis (OY) shows the classification outcomes for individual appliances (from top to bottom): dishwasher, tumble dryer, washing machine, and kettle. At the very bottom of each plot, information on abnormal behaviour of the electricity meters is indicated. For each appliance, up to four types of markers may appear (from top to bottom): missed detections (FN, blue), false positives (FP, red), correct detections (TP, green), and reference labels from the ground-truth devices (grey).

**Figure 4:**
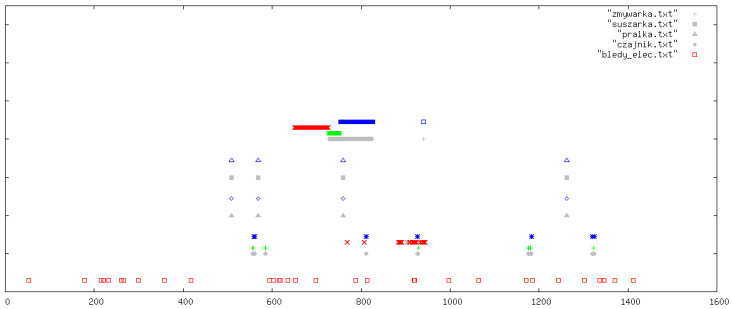
Example detections for another representative day. The horizontal axis (OX) represents time in minutes from the beginning of the day. The vertical axis (OY) shows the classification outcomes for individual appliances (from top to bottom): dishwasher, tumble dryer, washing machine, and kettle. At the very bottom of each plot, information on abnormal behaviour of the electricity meters is indicated. For each appliance, up to four types of markers may appear (from top to bottom): missed detections (FN, blue), false positives (FP, red), correct detections (TP, green), and reference labels from the ground-truth devices (grey).

The washing machine and dishwasher detectors achieved high specificity, while kettle detection was sensitive to noise and load overlap. Overall, multimodality strongly benefited devices with water signatures, stabilizing detections against purely electrical fluctuations.

Beyond the aggregate counts, inspection of individual days reveals several recurring patterns. For the washing machine, missed detections tend to occur in atypical cycles with shortened or interrupted rinses, or in cases where the cycle is split across day boundaries so that the required number of fills or drum-rotation phases is not observed within a single 24 h window. False alarms are virtually absent, reflecting the conservative design of the rules. For the dishwasher, most false positives appear in periods where other loads produce similar thermal profiles, emphasizing the importance of water information when available. In contrast, kettle detections illustrate the limits of this dataset: nonlinearity of the electrical meter and concurrency with other devices can easily distort short transients, making it difficult to balance sensitivity and specificity simultaneously for this appliance.

**Figure 5:**
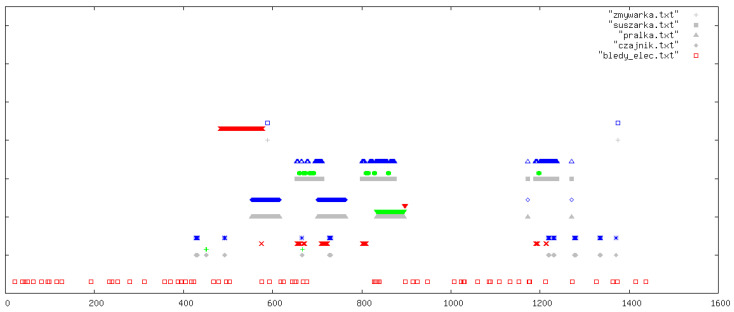
Example detections: focus on washing machine and dishwasher activity. The horizontal axis (OX) represents time in minutes from the beginning of the day. The vertical axis (OY) shows the classification outcomes for individual appliances (from top to bottom): dishwasher, tumble dryer, washing machine, and kettle. At the very bottom of each plot, information on abnormal behaviour of the electricity meters is indicated. For each appliance, up to four types of markers may appear (from top to bottom): missed detections (FN, blue), false positives (FP, red), correct detections (TP, green), and reference labels from the ground-truth devices (grey).

**Figure 6:**
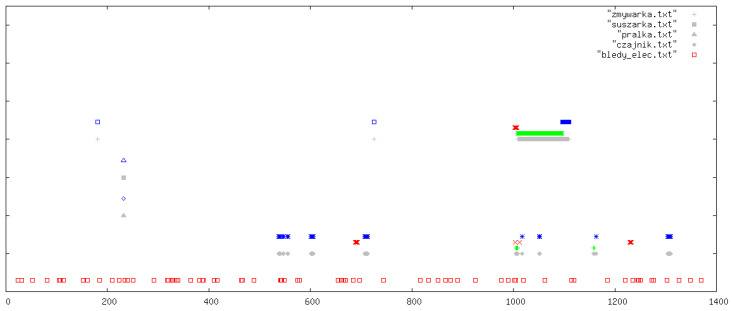
Example detections: focus on tumble dryer and kettle activity. The horizontal axis (OX) represents time in minutes from the beginning of the day. The vertical axis (OY) shows the classification outcomes for individual appliances (from top to bottom): dishwasher, tumble dryer, washing machine, and kettle. At the very bottom of each plot, information on abnormal behaviour of the electricity meters is indicated. For each appliance, up to four types of markers may appear (from top to bottom): missed detections (FN, blue), false positives (FP, red), correct detections (TP, green), and reference labels from the ground-truth devices (grey).

From a methodological point of view, these observations suggest that summary statistics alone are insufficient to characterize detector performance. The extended set of daily plots in the Appendix A serves as a qualitative complement to the numeric results, allowing readers to judge how the rules behave in edge cases and to what extent particular errors are understandable or acceptable in a deployment scenario.

**Figure 7:**
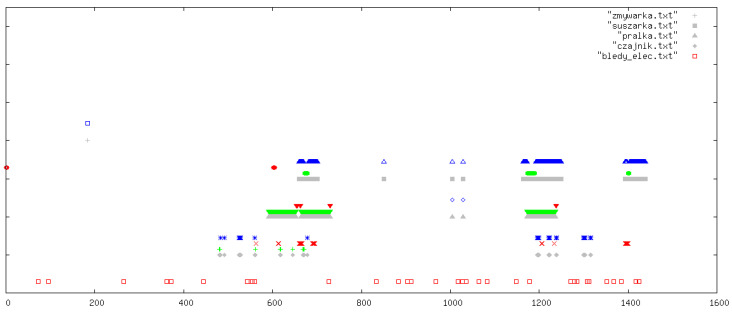
Example detections for a day with multiple overlapping appliance events. The horizontal axis (OX) represents time in minutes from the beginning of the day. The vertical axis (OY) shows the classification outcomes for individual appliances (from top to bottom): dishwasher, tumble dryer, washing machine, and kettle. At the very bottom of each plot, information on abnormal behaviour of the electricity meters is indicated. For each appliance, up to four types of markers may appear (from top to bottom): missed detections (FN, blue), false positives (FP, red), correct detections (TP, green), and reference labels from the ground-truth devices (grey).

The verification experiments were carried out on data collected and supplied by Vedia SA. As already mentioned, reliable reference information was available for four devices: washing machine, dishwasher, tumble dryer, and kettle. Data were measured at the level of total household consumption, and it must be remembered that other appliances were active in the dwelling. In particular, devices for which the reference logging failed (iron, vacuum cleaner, microwave oven) and large appliances without reference sensors (refrigerator, electric hob, oven) all contribute to the aggregate signal and can interfere with the studied detectors. The power drawn by these additional appliances is comparable to that of the four analysed devices and can therefore mask or distort their signatures.

Figures in the main text cover five representative days, while the Appendix A presents plots for some examples of the remaining days. Missing days usually correspond to periods where measurement problems prevented reliable data collection. All plots use the same conventions for axes and colours as described earlier.

Based on the full set of daily plots, the following qualitative summary can be offered. For the washing machine, 22 out of 30 recorded cycles were successfully detected. In three additional cases the algorithm would have detected the cycle, but returned a negative result due to the strict requirement of observing at least two rinses and a complete spin phase within the same day; these were classified as misses (FN) rather than false positives. In total, eight washing-machine cycles were missed and none were falsely declared when they did not occur. For the dishwasher, 19 out of 21 cycles were detected, though the precise start and end times were not always perfectly aligned with the reference. Five false positives were observed, typically coinciding with periods where other large loads were active, and two reference cycles were missed. For the tumble dryer, 23 out of 27 drying sessions produced at least a partial detection of drum rotation; there were four misses and no false positives, with the caveat that we count partial detections as successes. For the kettle, a cluster-based reading of the daily plots yields approximately 61 true positives, 72 false positives, and 150 false negatives, illustrating again that this appliance is the most challenging in this dataset.

## 6. Discussion

Rule-based detectors proved viable for real multimodal data but remain dependent on sensor fidelity and label quality. Multimodal signals substantially improved recognition of washer and dishwasher cycles, as the joint electricity–water behaviour reduced ambiguity. The kettle remained challenging due to short, high-power transients confounded by nonlinear meter response and coincident loads.

Compared to purely learning-based methods, our approach is interpretable, lightweight, and installation-friendly. Its transparency makes it suitable for low-cost or privacy-sensitive deployments. However, tuning thresholds manually limits portability between households. Future systems may incorporate automated parameter estimation or adaptive thresholds derived from small calibration samples.

Hybrid approaches combining rule-based priors with machine learning adaptors could integrate the best of both paradigms: explainable logic where feasible, and data-driven correction where necessary. In particular, the present detectors could serve as candidates for weak labels or priors in semi-supervised NILM pipelines.

A further aspect concerns user trust and accountability. In many smart metering scenarios, NILM outputs may eventually be used not only for feedback but also for billing adjustments, efficiency incentives, or even contractual obligations. In such contexts, the ability to justify why a given event was detected or missed is not a cosmetic feature but a functional requirement. Rule-based components, with explicitly documented thresholds and temporal constraints, provide a natural audit trail that is difficult to obtain from purely black-box models.

The results also highlight the importance of choosing and interpreting performance measures in a way that matches stakeholder priorities. In this project, the decisive criterion for “better” was not a single global metric but the subjective assessment by domain experts (from Vedia SA) of daily activity plots. Given the imbalance of the problem and the imperfections of the data, this emphasis on visual interpretability proved more informative than raw accuracy figures. At the same time, the experience gathered here suggests directions for defining more nuanced, task-specific quantitative scores that explicitly penalize certain error types more heavily than others.

Finally, the case study illustrates that even a relatively small, single-household dataset can be valuable if it is documented with sufficient care. By exposing imperfections rather than hiding them, the study offers a realistic benchmark for future methods that aim to be robust in the wild. We expect that hybrid NILM architectures will evolve toward multi-layer designs in which simple detectors like those presented here co-exist with adaptive, data-driven components operating under explicit operational constraints.

Looking ahead, an important line of work is the automatic tuning of detector parameters via machine learning. To make such tuning meaningful, one needs an evaluation measure that reflects practical utility, or at least a reliable procedure for comparing two parameter sets and deciding which one is preferable. The present study, with its combination of scalar metrics and visual inspection, offers a starting point for defining such measures. Another related direction is the systematic construction of behavioural models for additional appliances: understanding which features of a device’s operation are necessary, which are optional, and how much variability can be tolerated without sacrificing specificity. Depending on how these choices are made, one can deliberately shift the balance between sensitivity (ability to detect as many true events as possible) and specificity (ability to avoid confusing the device with others).

Several simple strategies for adjusting this balance can already be identified from our experience. Sensitivity can be increased by relaxing some conditions (e.g., allowing a certain fraction of expected patterns to be missing, as in the washing-machine detector) or by adding alternative behavioural scenarios joined by logical “or” conditions. Specificity, in turn, can be improved by tightening value ranges and adding additional, independent conditions that must all be satisfied (logical “and”). In future work, these qualitative design choices could be systematically explored and combined with automated search over parameter spaces.

An additional methodological aspect concerns the sensitivity of the proposed detectors to parameter choices. In the case of rule-based systems, sensitivity can be assessed conceptually by perturbing key thresholds and temporal constraints (e.g., power levels, time windows, or persistence conditions) and observing the stability of detection outcomes. A systematic sensitivity analysis is beyond the scope of the present case study; however, the conservative design philosophy adopted here—favoring specificity over sensitivity—was intended to limit the impact of small parameter variations under realistic sensing conditions.

## 7. Conclusions

The reported identification accuracy should be interpreted in light of the deliberately conservative design choices adopted in this study. The proposed approach was not intended to maximize accuracy in a benchmark setting, but to provide simple and interpretable information under fully aggregated sensing conditions with uncontrolled background activity. For this reason, direct comparison with learning-based or benchmark-optimized NILM methods is outside the scope of the present work.

We validated transparent, rule-based detectors for four appliances using real-world multimodal household data (electricity, water, gas) measured at the level of total building consumption, with multiple other uninstrumented appliances and activities running concurrently. Despite common sensing imperfections (timestamp discontinuities, spurious zeros, and meter nonlinearity), the multimodal rules achieved high specificity for washing machine, dishwasher, and tumble dryer recognition (22/30, 19/21, and 23/27 detected cycles, respectively), while the kettle remained the most challenging device due to short transients and frequent overlap with other loads. Overall, the case study demonstrates that simple, explainable rules can still provide actionable detections in realistic deployments where idealized assumptions do not hold.

The main limitations of the study are the single-household scope, household-specific parameter tuning, and incomplete or imperfect reference labeling for some devices. In addition, our goal was not a benchmark comparison against the broader NILM literature but a documented validation of an interpretable scheme under non-ideal sensing conditions; therefore, results should be interpreted as a realistic baseline rather than as a claim of superiority over learning-based approaches. Sensitivity of the detectors can be assessed in future work by perturbing key thresholds and temporal parameters (e.g., power steps, minimum durations, and modality consistency windows) and measuring the stability of detections and error counts.

Future work will extend the dataset and evaluation to additional households and appliances, and will explore hybrid pipelines in which rule-based detectors provide interpretable priors or weak labels for learning-based components. Such designs may improve generalization while retaining transparency and operational constraints suitable for practical energy management systems. 

## Figures and Tables

**Table 1:** Illustrative results from a previous feature-based experiment showing the limitations of scalar metrics alone.

Device	Accuracy	Sensitivity
Washing machine	68%	(machine inactive on that day)
Dishwasher	66%	77%
Kettle	99%	0%

**Figure A1:**
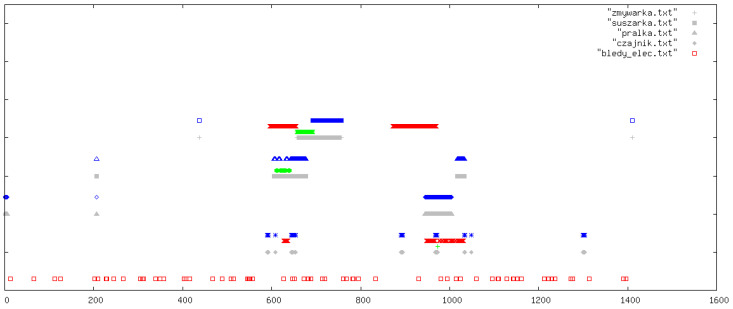
Example of day 1. For each appliance, up to four types of markers may appear: missed detections (FN, blue), false positives (FP, red), correct detections (TP, green), and reference labels from the ground-truth devices (grey).

**Figure A2:**
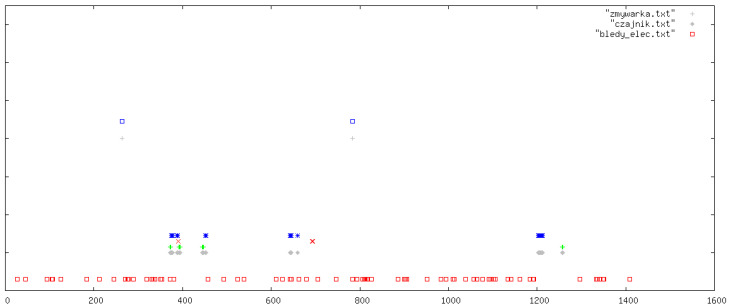
Example of day 2. For each appliance, up to four types of markers may appear: missed detections (FN, blue), false positives (FP, red), correct detections (TP, green), and reference labels from the ground-truth devices (grey).

**Figure A3:**
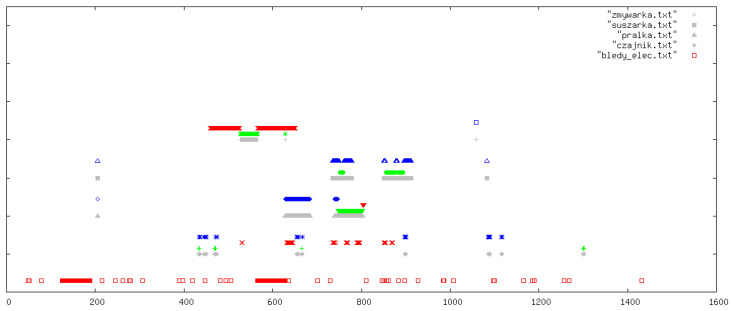
Example of day 3. For each appliance, up to four types of markers may appear: missed detections (FN, blue), false positives (FP, red), correct detections (TP, green), and reference labels from the ground-truth devices (grey).

**Figure A4:**
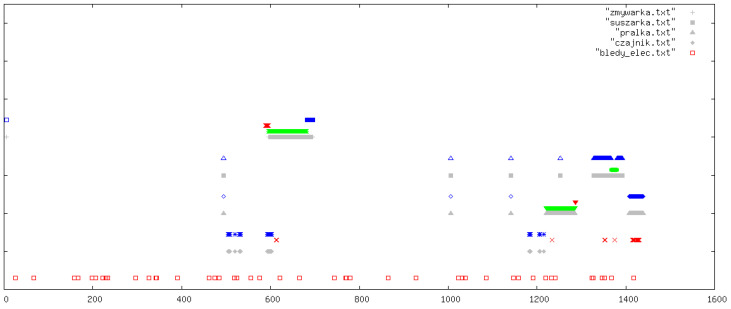
Example of day 4. For each appliance, up to four types of markers may appear: missed detections (FN, blue), false positives (FP, red), correct detections (TP, green), and reference labels from the ground-truth devices (grey).

**Figure A5:**
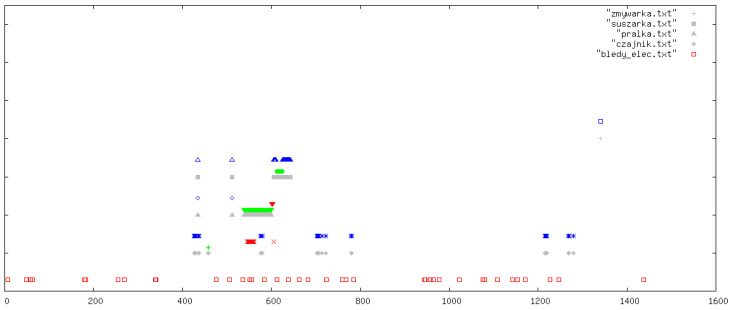
Example of day 5. For each appliance, up to four types of markers may appear: missed detections (FN, blue), false positives (FP, red), correct detections (TP, green), and reference labels from the ground-truth devices (grey).

**Figure A6:**
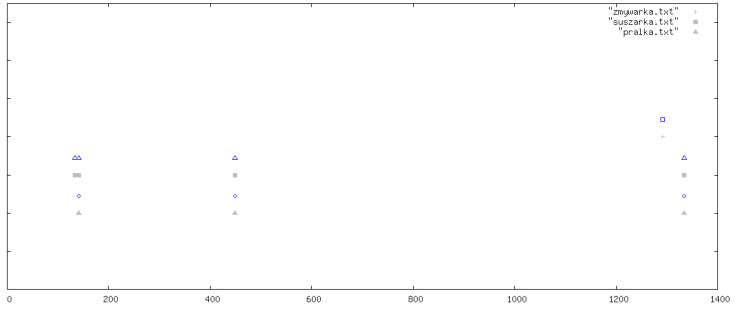
Example of day 6. For each appliance, up to four types of markers may appear: missed detections (FN, blue), false positives (FP, red), correct detections (TP, green), and reference labels from the ground-truth devices (grey).

**Figure A7:**
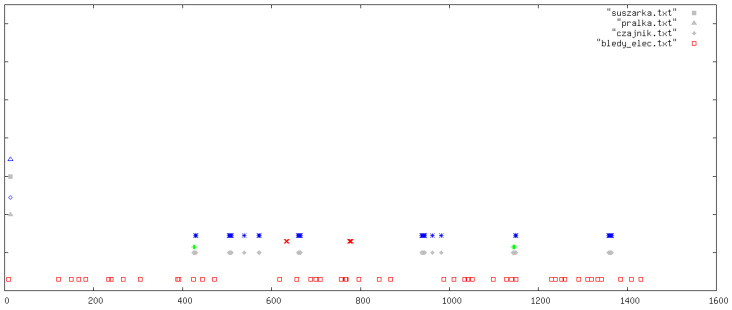
Example of day 7. For each appliance, up to four types of markers may appear: missed detections (FN, blue), false positives (FP, red), correct detections (TP, green), and reference labels from the ground-truth devices (grey).

**Figure A8:**
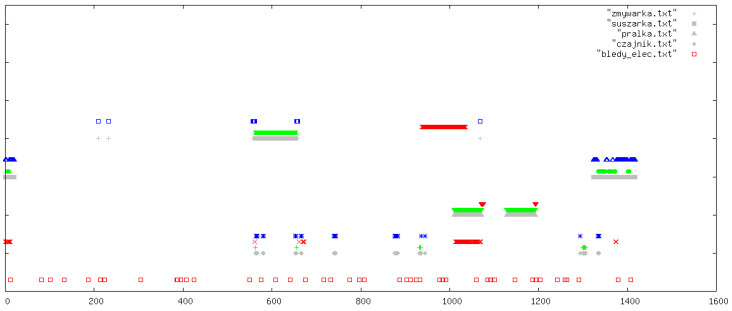
Example of day 8. For each appliance, up to four types of markers may appear: missed detections (FN, blue), false positives (FP, red), correct detections (TP, green), and reference labels from the ground-truth devices (grey).

**Figure A9:**
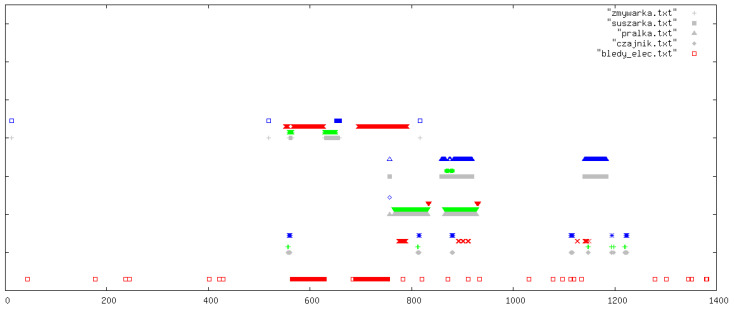
Example of day 9. For each appliance, up to four types of markers may appear: missed detections (FN, blue), false positives (FP, red), correct detections (TP, green), and reference labels from the ground-truth devices (grey).

**Figure A10:**
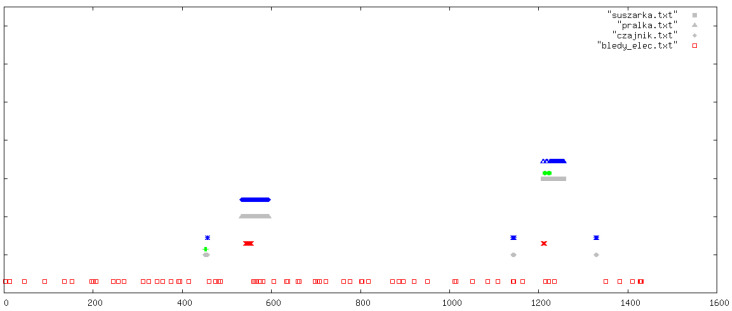
Example of day 10. For each appliance, up to four types of markers may appear: missed detections (FN, blue), false positives (FP, red), correct detections (TP, green), and reference labels from the ground-truth devices (grey).

**Figure A11:**
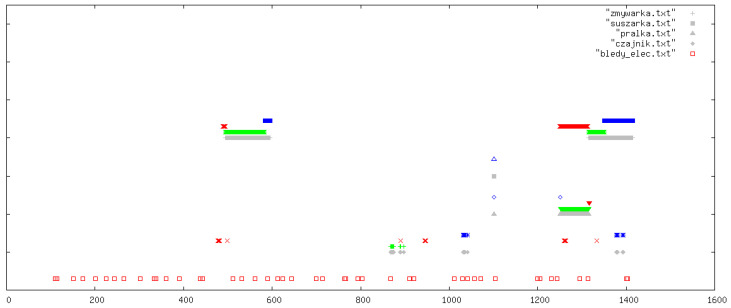
Example of day 11. For each appliance, up to four types of markers may appear: missed detections (FN, blue), false positives (FP, red), correct detections (TP, green), and reference labels from the ground-truth devices (grey).

**Figure A12:**
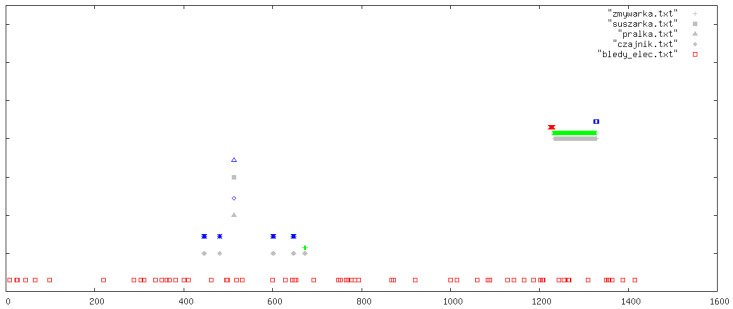
Example of day 12. For each appliance, up to four types of markers may appear: missed detections (FN, blue), false positives (FP, red), correct detections (TP, green), and reference labels from the ground-truth devices (grey).

**Figure A13:**
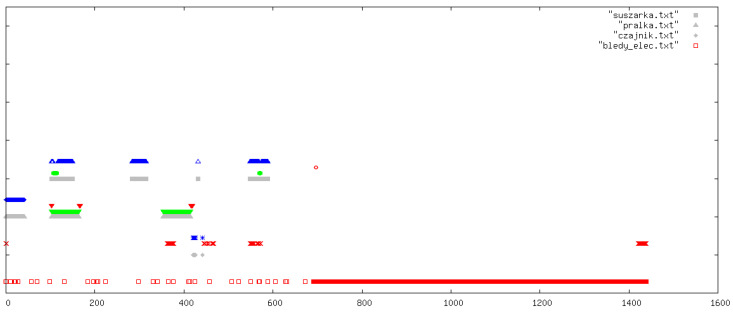
Example of day 13. For each appliance, up to four types of markers may appear: missed detections (FN, blue), false positives (FP, red), correct detections (TP, green), and reference labels from the ground-truth devices (grey).

## Data Availability

The original contributions presented in this study are included in the article material. Further inquiries can be directed to the corresponding author.

## Appendix A. Extended Detection Examples

The plots given here are a continuation of examples from Figure 5, Figure 6 and Figure 7 and present another 13 days of measurements. As in the previous case, the horizontal axis (OX) represents time in minutes from the beginning of the day. The vertical axis (OY) shows the classification outcomes for individual appliances (from top to bottom): dishwasher, tumble dryer, washing machine, and kettle. At the very bottom of each plot, information on abnormal behaviour of the electricity meters is indicated. For each appliance, up to four types of markers may appear (from top to bottom): missed detections (FN, blue), false positives (FP, red), correct detections (TP, green), and reference labels from the ground-truth devices (grey).
